# Extricating New Physics Scenarios at DUNE with Higher Energy Beams

**DOI:** 10.1038/s41598-018-36790-6

**Published:** 2019-01-23

**Authors:** Mehedi Masud, Mary Bishai, Poonam Mehta

**Affiliations:** 1 Astroparticle and High Energy Physics Group, Institut de Física Corpuscular – C.S.I.C./Universitat de València, Parc Cientific de Paterna., C/Catedratico José Beltrán, 2E-46980 Paterna, València Spain; 2 Harish-Chandra Research Institute, Chattnag Road, Allahabad, 211 019 India; 3 Brookhaven National Laboratory, P.O. Box 5000, Upton, NY 11973 USA; 4 School of Physical Sciences, Jawaharlal Nehru University, New Delhi, 110067 India

## Abstract

The proposed Deep Underground Neutrino Experiment (DUNE) utilizes a wide-band on-axis tunable muon-(anti)neutrino beam with a baseline of 1300 km to search for CP violation with high precision. Given the long baseline, DUNE is also sensitive to effects due to matter induced non-standard neutrino interactions (NSI) which can interfere with the standard three-flavor oscillation paradigm. Hence it is desirable to design strategies to disentangle effects due to NSI from standard oscillations. In this article, we exploit the tunability of the DUNE neutrino beam over a wide-range of energies to devise an experimental strategy for separating oscillation effects due to NSI from the standard three-flavor oscillation scenario. Using *χ*^2^ analysis, we obtain an optimal combination of beam tunes and distribution of run times in neutrino and anti-neutrino modes that would enable DUNE to isolate new physics scenarios from the standard. We can distinguish scenarios at 3*σ* (5*σ*) level for almost all (~50%) values of *δ*. To the best of our knowledge, our strategy is entirely new and has not been reported elsewhere.

## Introduction

Neutrino oscillations among the three flavours have been firmly established and the experimental confirmation of neutrino oscillations vindicates that the Standard Model (SM) of particle physics is incomplete^1^. The minimal extension of SM invokes a mechanism to generate tiny neutrino masses while retaining the interactions as predicted in the SM. We refer to this minimal model as Standard Interactions (SI).

Most of the parameters responsible for standard three flavor neutrino oscillations have been measured with fairly good precision except for a few^2^. Some of the yet unresolved questions in neutrino physics include whether CP is violated, if the neutrino mass hierarchy is normal or inverted and what the correct octant of *θ*_23_ is. Ascertaining violation or conservation of leptonic CP invariance is one of the most challenging goals in particle physics, astrophysics and cosmology. Whatever the answer would be, it will have crucial bearing upon the bigger question of why there is more matter than antimatter in the Universe. In the quark sector, CP violation has been experimentally measured and within the SM, it originates from the single phase in the 3 × 3 mixing matrix (commonly known as the Cabibbo Kobayashi Maskawa (CKM) matrix). In the leptonic sector, the three active neutrinos have masses and mix. Therefore, one expects a CP violating phase to appear in the 3 × 3 leptonic mixing matrix (usually referred to as the Pontecorvo Maki Nakagawa Sakata (PMNS) matrix) as well.

The future long baseline accelerator experiments such as Deep Underground Neutrino Experiment (DUNE)^3^ (and also Tokai to HyperKamiokande (T2HK) in Japan) are planned in such a way that they present an excellent opportunity to decipher whether CP is violated in the leptonic sector. Further, if the answer to the question posed is in affirmative, one would like to measure the value of the CP phase (*δ*) with reasonable precision. DUNE and the facility that will support it, the Long-Baseline Neutrino Facility (LBNF), will be an internationally designed, coordinated and funded program, hosted at the Fermi National Accelerator Laboratory (Fermilab) in Batavia, Illinois^4^.

With this backdrop, let us also mention that a clean measurement of CP phase is a herculean task. The reason is simple, in case of any long baseline experiment, neutrinos traverse matter and ordinary matter effects in SI introduce extrinsic CP contribution (matter being CP asymmetric) which obscures the determination of the intrinsic CP phase (appearing in the mixing matrix). In the presence of new physics effects, clean extraction of the CP violating phase becomes a formidable task^5–7^. In fact, a given measured value of CP phase could very well be a hint of new physics^8,9^. In earlier works, it has been pointed out that there are degeneracies within the large parameter space in the presence of non-standard interactions (NSI)^10–24^. The need to devise ways to distinguish between the standard paradigm and new physics scenarios has been extensively discussed (for other new physics scenarios, see for example^25,26^ and references therein).

**Figure 1:**
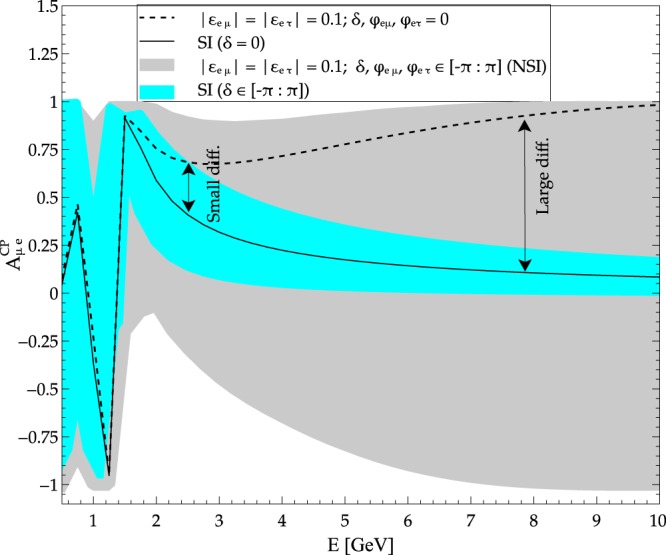
CP asymmetry plotted as a function of energy for a baseline of 1300 km relevant for DUNE. The solid (dashed) black curve is for SI case with *δ* = 0 (NSI case with non-zero moduli but zero phases). The cyan band is for SI with *δ* taking all possible values in the allowed range. The grey band is for NSI with all phases in allowed ranges.

To illustrate the impact of new physics scenario such as NSI considered in the present work, let us examine Fig. 1 in which the CP asymmetry in the ${\nu }_{\mu }\to {\nu }_{e}$ channel computed for the baseline of 1300 km relevant to DUNE for SI and NSI. The choice of NSI parameters is only a *representative choice* consistent within present constraints to qualitatively assess the impact of NSI. It should be noted that difference between SI and NSI increases as we go to higher energies beyond ($E\gtrsim 5\,{\rm{GeV}}$) in Fig. 1. Note that this was first illustrated in one of our previous works^12^. The standard beam used by DUNE is peaked around the first oscillation maximum (~2.5 GeV) and this basically reduces the observability of the otherwise large difference between SI and NSI at higher energies. This calls for a need to strategise for harnessing the large difference in CP asymmetries for the two considered scenarios at larger values of energy. Fortituiously, the beam considered for DUNE is a wide band beam and allows for tunability which allows for other beam options with significant flux at higher energies than the standard beam used thus far in all the existing studies. We exploit the tunability of the beam and offer a strategy which could lead to better identification and discrimination of the new physics effects. Precisely, this particular observation gave us the insight of utilitizing the higher energy beams for the purpose of isolating physics scenarios.

In a novel approach, we use experimental handles that could prove useful to differentiate between the standard scenario (with only one source of CP violation) and new physics scenario (which inevitably brings in more parameters including new sources of CP violating phases). We propose a *χ*^2^ quantity which is our theoretical metric that allows us to optimize experimental strategies and combine beam tunes for the purpose of distinguishing NSI from SI. We finally deduce optimal beamtune and runtime combination to achieve this goal. Recent studies have explored the sensitivities to SI parameters and the synergies between experiments (including DUNE) using different baselines and neutrino beam energies^27^ (see also^28^). In this study, for the first time, we explore sensitivities to distinguish SI and NSI effects at a fixed baseline over a large range of *L*/*E* using *DUNE’s unique broad-band tunable beam*. Different wide-band fluxes can be experimentally achieved using the DUNE NuMI-style reference beam design^4^ by simply varying the target and horn placement^29^.

The plan of the article is as follows. We begin with a brief description of the model of new physics i.e., NSI considered in the present work in Section 2. In Section 3, we describe the neutrino beam tunes considered in the present work in the context of DUNE. Section 4 is devoted to explaining the numerical procedure followed in the present work. We report our findings along with discussion in Section 5. Finally, we conclude in Section 6.

## Non-Standard Neutrino Interaction Model

The effective Hamiltonian in the flavour basis entering the Schrödinger equation for neutrino propagation is given by

1 $$\begin{array}{rcl}{ {\mathcal H} }_{{\rm{f}}} & = & { {\mathcal H} }_{{\rm{v}}}+{ {\mathcal H} }_{{\rm{SI}}}+{ {\mathcal H} }_{{\rm{NSI}}}\\  & = & \lambda \{{\mathscr{U}}(\begin{array}{ccc}0 &  & \\  & {r}_{\lambda } & \\  &  & 1\end{array}){{\mathscr{U}}}^{\dagger }+{r}_{A}(\begin{array}{ccc}1 & 0 & 0\\ 0 & 0 & 0\\ 0 & 0 & 0\end{array})+{r}_{A}(\begin{array}{ccc}{\varepsilon }_{ee} & {\varepsilon }_{e\mu } & {\varepsilon }_{e\tau }\\ {\varepsilon }_{e\mu }^{\ast } & {\varepsilon }_{\mu \mu } & {\varepsilon }_{\mu \tau }\\ {\varepsilon }_{e\tau }^{\ast } & {\varepsilon }_{\mu \tau }^{\ast } & {\varepsilon }_{\tau \tau }\end{array})\},\end{array}$$

The three terms in Eq. 1 are due to vacuum $({ {\mathcal H} }_{{\rm{v}}})$, matter with SI $({ {\mathcal H} }_{{\rm{SI}}})$ and matter with NSI $({ {\mathcal H} }_{{\rm{NSI}}})$ respectively. For the NSI case, the ${\varepsilon }_{\alpha \beta }\,(\,\equiv \,|{\varepsilon }_{\alpha \beta }|\,{e}^{i{\phi }_{\alpha \beta }})$ are complex parameters which appear in ${ {\mathcal H} }_{NSI}$. The ratios *λ*, *r*_*λ*_ and *r*_*A*_ appearing in Eq. 1 are

2 $$\lambda \equiv \frac{\delta {m}_{31}^{2}}{2E};\,{r}_{\lambda }\equiv \frac{\delta {m}_{21}^{2}}{\delta {m}_{31}^{2}};\,{r}_{A}\equiv \frac{A(x)}{\delta {m}_{31}^{2}}.$$

and $A(x)=2\sqrt{2}E{G}_{F}{n}_{e}(x)$ where *G*_*F*_ is the Fermi constant, *E* is the energy, *n*_*e*_(*x*) is the electron number density. The mass-squared difference is given by $\delta {m}_{ij}^{2}={m}_{i}^{2}-{m}_{j}^{2}$. ${\mathscr{U}}$ is the 3 × 3 mixing matrix which in the commonly used PMNS parameterization is given by

$$\begin{array}{ccc}{\mathscr{U}} & = & (\begin{array}{lll}{c}_{12}{c}_{13} & {s}_{12}{c}_{13} & {s}_{13}{e}^{-i\delta }\\ -{s}_{12}{c}_{13}-{c}_{12}{s}_{13}{s}_{23}{e}^{i\delta } & {c}_{12}{c}_{23}-{s}_{12}{s}_{13}{s}_{23}{e}^{i\delta } & {c}_{13}{s}_{23}\\ {s}_{12}{s}_{23}-{c}_{12}{s}_{13}{c}_{23}{e}^{i\delta } & -{c}_{12}{s}_{23}-{s}_{12}{s}_{13}{c}_{23}{e}^{i\delta } & {c}_{13}{c}_{23}\end{array})\end{array},$$

where ${s}_{ij}=\,\sin \,{\theta }_{ij},{c}_{ij}=\,\cos \,{\theta }_{ij}$ and *δ* is the Dirac-type CP phase. The two additional Majorana-type phases play no role in neutrino oscillations and hence omitted.

As a result of the hermiticity of the Hamiltonian, we have nine additional parameters (three phases and six amplitudes appearing ${ {\mathcal H} }_{NSI}$). Thus, there are new genuine sources of CP violation as well as new fake sources of CP violation (aka matter effects) that can change the CP asymmetries even further. For more details, see^12,16,18^ and references therein.

## Neutrino Beam Tunes

**Figure 2:**
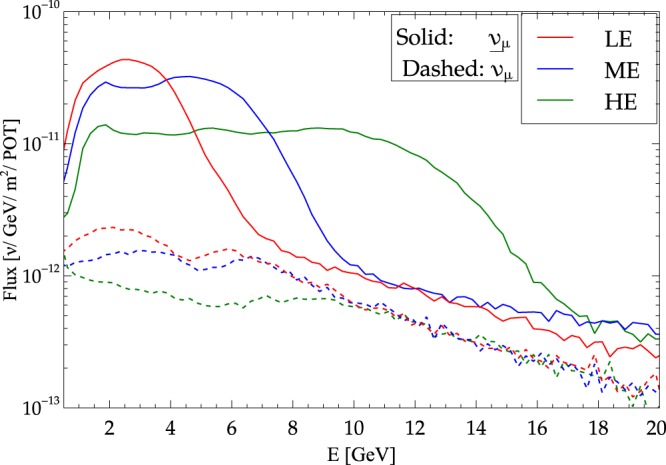
Comparison of the different flux tunes (LE, ME, HE) in the neutrino running mode. POT stands for protons on target.

**Figure 3:**
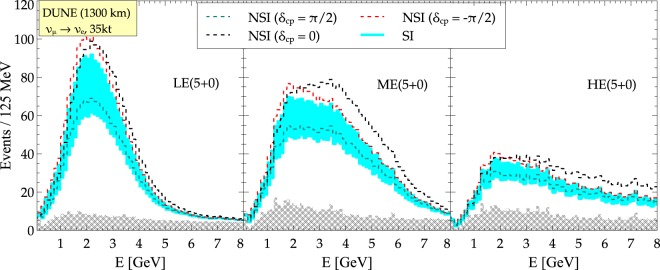
Separation between SI ${\nu }_{\mu }\to {\nu }_{e}$ events (cyan band, red and magenta dashed lines) and NSI ${\nu }_{\mu }\to {\nu }_{e}$ events (black dashed lines) at DUNE with LE (5 + 0), ME (5 + 0) and HE (5 + 0) beam tunes. The black dashed line is for a CP conserving NSI scenario. The cyan band corresponds to the SI case with the full variation of *δ*. The dashed lines are for NSI case with different true values of *δ*. The background events are similar for all the four cases and are shown as grey shaded region.

The standard neutrino flux (referred to as low energy (LE) beam) is peaked at energy values close to the first oscillation maximum (*E* ~ 2.5 GeV for DUNE) (see Fig. 2). So, when the large CP asymmetry prominent at higher energies at the probability level is folded with the standard LE flux to generate the events, the difference between standard and new physics is masked because of the falling flux (this can be seen from the leftmost plot in Fig. 3, also note that this was pointed out for the first time in^12^.). It is therefore worthwhile and timely to ask if we can suitably tap the large signal of CP asymmetry at higher energies using higher energy beams.

For this study, we considered three wide-band beam tunes obtained from a full Geant4 simulation^30,31^ of a neutrino beamline using NuMI-style focusing. The beam tunes considered are: LE; medium energy (ME); and high energy (HE) as shown in Fig. 2. These beam tunes are consistent with what could be achieved by the LBNF facility. The beamline parameters assumed for the different design fluxes used in our sensitivity calculations are given in Table 1 (see^32,33^).

**Table 1:** Beamline parameters assumed for the different design fluxes used in our sensitivity calculations^32,33^.

Parameter	LE	ME	HE
Proton Beam	*E*_*p*+_ = 120/80 GeV, 1.2–2.4 MW		
Focusing	2 NuMI horns, 230 kA, 6.6 m apart		
Target location	−25 cm	−1.0 m	−2.5 m
Decay pipe length	250 m	250 m	250 m
Decay pipe diameter	4 m	4 m	4 m

The target is a thin Be cylinder 2 interaction lengths long. The target location is given with respect to the upstream face of Horn 1. The LBNF neutrino beamline decay pipe length has been chosen to be 194 m. Decay pipe lengths of up to 250 m could be accommodated on the Fermilab site and were an option in previous designs of the beamline.

## Numerical Procedure

To quantify the separation of physics scenarios (SI-NSI separation), we define the (statistical) *χ*^2^ as follows -

3 $${\chi }^{2}({\delta }_{tr})=\mathop{{\rm{\min }}}\limits_{{\delta }_{ts}}\,\sum _{i=1}^{x}\,\sum _{j}^{2}\,\frac{{[{N}_{NSI}^{i,j}({\delta }_{tr},|{\varepsilon }_{\alpha \beta }|,{\phi }_{\alpha \beta })-{N}_{SI}^{i,j}({\delta }_{ts}\in [-\pi ,\pi ])]}^{2}}{{N}_{NSI}^{i,j}({\delta }_{tr},|{\varepsilon }_{\alpha \beta }|,{\phi }_{\alpha \beta })}$$

where, ${N}_{SI}^{i,j}$ and ${N}_{NSI}^{i,j}$ are the number of events in the {*i*, *j*}-th bin for the case of SI and NSI respectively^16^. Note that the definition of the *χ*^2^ in Eq. 3 includes only statistical effects and facilitates our understanding. The systematic effects are taken into account in the numerical results. NSI parameters are expressed in terms of moduli $|{\varepsilon }_{\alpha \beta }|$ and phases *φ*_*αβ*_. *δ*_*ts*_(*δ*_*tr*_) is the test (true) value of *δ*. The index *i* runs over the number of energy bins for a given experiment. The energy range for DUNE is *E* = 0–20 GeV and we have a binned energy spectrum. Note that we have a total of 71 bins (*x* = 71 in Eq. 3) of non-uniform bin widths (64 bins with uniform bin width of 125 MeV in the energy range *E* = 0–8 GeV and variable bin width beyond 8 GeV)^33^. The sum over *j* runs over neutrino and antineutrinos for a given channel. We utilise both ${\nu }_{\mu }\to {\nu }_{e}$ (appearance) and ${\nu }_{\mu }\to {\nu }_{\mu }$ (disappearance) channels. We assume that all SI parameters (except *δ*) are well-determined and therefore we marginalise over *δ*_*ts*_ only. This *χ*^2^ was calculated using a set of conservative and fixed choice of the non-zero NSI parameters ($|{\varepsilon }_{e\mu }|$ = 0.04, $|{\varepsilon }_{e\tau }|$ = 0.04 *ε*_*ee*_ = 0.4)^34,35^. For the sake of simplicity, the NSI phases are set to zero. We also discuss the impact of non-zero NSI phases towards the end.

We have implemented a GLoBES^36,37^ simulation of a 1300 km baseline neutrino beam experiment using a parameterization of the DUNE far detector response as described in^33^. We assume normal hierarchy (NH) in all the plots.

## Results and Discussion

We now discuss the impact of using different beam tunes and run time combinations on the separability of physics scenarios.

### Impact of beam tunes on the event spectrum

We show the variation in the ${\nu }_{e}$ event spectrum in Fig. 3 for the LE, ME and HE beam tunes under SI and NSI scenarios. For all the beam tunes, the red dashed line corresponds to *δ* = −*π*/2 with NSI, green dashed line corresponds to *δ* = +*π*/2 with NSI and the cyan band is for SI for *δ* ∈ [−*π*, *π*]. The backgrounds are shown as grey shaded region. The black dashed lines (for *δ* = 0 with NSI) lie farthest apart from the cyan band (SI) which means that one expects better separability between the two considered scenarios at values of *δ* ~ 0 (or ±*π*). In addition, even though the total events in ME or HE only case are smaller than that in the LE only case, better separation between SI and NSI scenarios can be achieved if we can make use of the ME or HE beam.

### Impact of beam tunes on sensitivity to CP violation

**Figure 4:**
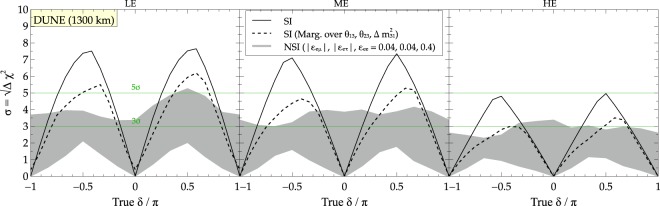
CP violation sensitivity for the three beam tunes for run time of $5\nu +5\bar{\nu }$ years for SI (black solid line) and NSI case (grey band). The solid black line depicts marginalization *δ* only while the dashed black line depicts marginalisation carried out over other oscillation parameters (${\theta }_{23},{\theta }_{13},\delta {m}_{31}^{2}$) as well.

In Fig. 4, the sensitivity to CP violation using appearance and disappearance channels (for more details, see^12,16^) is depicted using three different fluxes for a run time of $5\nu +5\bar{\nu }$ years for SI and NSI cases respectively. The black solid curve is obtained by marginalizing over *δ* only. We note that in all the cases, the CP sensitivities drop (by almost 2*σ* near the peak) if we marginalize over other oscillation parameters (${\theta }_{23},{\theta }_{13},\delta {m}_{31}^{2}$) also. Nevertheless, among the different beam tunes, the LE beam tune seems to be our best bet for CP violation sensitivity if we consider the different available beam tunes in isolation. We then combine different beam tunes and vary runtimes to see if we can have an advantage towards answering the question that we have posed above (see Eq. (3)).

### Impact of beam tunes on extricating physics scenarios

**Figure 5:**
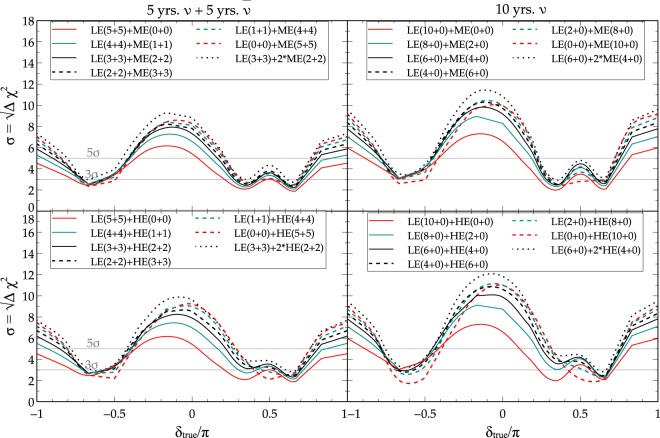
Separation between SI and NSI events at DUNE with different beam tunes at *χ*^2^ level. A CP conserving NSI scenario is assumed. The left column shows 5 years of neutrino and 5 years of anti-neutrino run times, while the right column depicts the case of 10 years of neutrino run time only.

In Fig. 5, we show the ability of DUNE to separate SI from NSI using different combinations of beam tunes and running times at the *χ*^2^ level (as a function of true *δ*). The left column is for an equal distribution of run time among neutrino and anti-neutrino modes while the right column corresponds to running in neutrino-only mode with the same total run time. A CP conserving NSI scenario is assumed in this plot (we assume ${\phi }_{e\mu }={\phi }_{e\tau }=0$). We have considered a combination of appearance (${\nu }_{\mu }\to {\nu }_{e}$) and disappearance (${\nu }_{\mu }\to {\nu }_{\mu }$) channels. The solid and dashed lines assume a beam power of 1.2 MW for both LE and ME beam tunes. The dotted black line corresponds to an ME option upgraded to 2.4 MW which is planned for later stages of DUNE. We note that the dominant channel contributing to the distinction of different physics scenarios is the ${\nu }_{\mu }\to {\nu }_{e}$ channel irrespective of our choice of the beam tune. The ${\nu }_{\mu }\to {\nu }_{\mu }$ channel adds somewhat (~1.5 − 2*σ* near the peak value at *δ* = 0) to the total sensitivity but the ${\nu }_{\mu }\to {\nu }_{\tau }$ contribution is negligible.

As noted earlier in the context of Fig. 3, we find that the ability to separate between the two scenarios tends to increase at CP conserving values of *δ* i.e. *δ* ~ 0, ±*π*. The dips seen near *δ* ~ ±*π*/2 (true) in Fig. 5 for combinations of LE and ME options in the top row or LE and HE options in the bottom row imply significantly smaller ability to distinguish between the scenarios particularly around those values of *δ*. In fact, in general, the different beam tunes and run time combinations other than LE only (solid red line) yield better results (see Fig. 5 and Table 2). From Fig. 5, we note that LE only is not the best choice for isolation of physics scenarios. And, the neutrino only mode gives somewhat better results. The above conclusions remain even if we choose the hierarchy to be inverted. An upgrade of beam power in ME (dotted black line) to 2.4 MW significantly improves the outcome.

**Table 2:** Approximately desirable combinations of beam tunes and runtimes deduced from Fig. 5 corresponding to maximizing the SI-NSI separation sensitivity assuming normal beam power 1.2 MW and with a total run time of 10 years.

5 year $\nu +5$ year $\bar{\nu }$	10 year $\nu +0$ year $\bar{\nu }$
LE (0 + 0) + ME (5 + 5)	LE (2 + 0) + ME (8 + 0)
LE (0 + 0) + HE (5 + 5)	LE (2 + 0) + HE (8 + 0)

### Impact of beam tunes on extricating physics scenarios via the fraction plots

**Figure 6:**
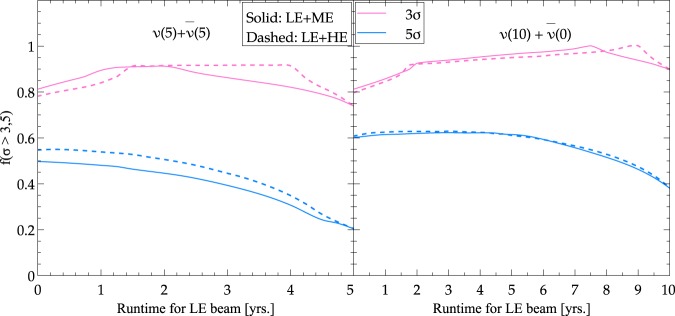
The fraction of the values of *δ* for which SI and NSI scenarios can be distinguished above 3*σ* (magenta) and 5*σ* (blue) using different combinations of beam tunes (LE + ME or LE + HE).

Another important factor driving the sensitivity to SI-NSI separation is the fraction of values of CP phase for which the sensitivity is more than 3*σ* or 5*σ*. In Fig. 6, the fraction of *δ* values for which the sensitivity lies above 3*σ* (magenta) and 5*σ* (blue) is plotted as a function of the run time for a combination of LE and ME (or HE) tuned beams. Both the panels are for a total run time of 10 years: the left one showing the case of 5 years of $\nu$ and 5 years of $\bar{\nu }$ run time while the right panel depicting the scenario of 10 years of $\nu$ run time alone. In the left panel, the 5 + 5 years of run time are distributed among the LE and ME (HE) beams for the solid (dashed) lines in the following manner: (*x* years of $\nu$ + *x* years of $\bar{\nu }$) of LE beam +((5 − *x*) + (5 + *x*)) years of ME or HE runtime. Similarly, the runtime in the right panel has been distributed as (*x* + 0) years of LE beam +((10 − *x*) + 0) years of ME or HE run time. We wish to stress that the fraction curves in Fig. 6 (see Table 3 for the desirable combination of beam tunes and runtimes deducted from Fig. 6) only show what portion of the sensitivity curve lies above 3*σ* (or 5*σ*), and not necessarily the absolute value of the sensitivities. The estimate of the fraction of *δ* values thus depends on the points of intersection of the sensitivity curve with the 3*σ* (or 5*σ*) horizontal lines in Fig. 5.

**Table 3:** Approximately desirable combinations of beam tunes and runtimes deduced from Fig. 6 corresponding to maximising the fraction of CP phase values above 3*σ* for SI-NSI separation as a function of run time in LE beam assuming normal beam power 1.2 MW and with a total run time of 10 years.

5 year $\nu +5$ year $\bar{\nu }$	10 year $\nu +0$ year $\bar{\nu }$
LE (2 + 2) + ME (3 + 3)	LE (7.5 + 0) + ME (2.5 + 0)
LE (2 + 2) + HE (3 + 3)	LE (9 + 0) + HE (1 + 0)

### Impact of beam tunes on separation between physics scenarios for CP nonconserving NSI

**Figure 7:**
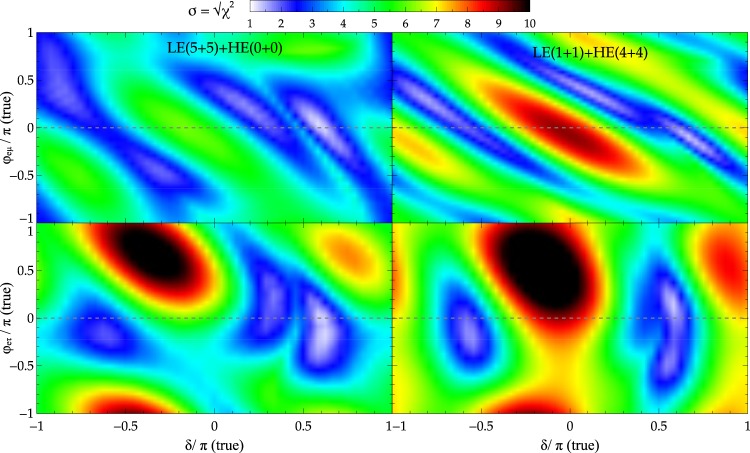
Effect of non-zero phases, *φ*_*eμ*_ (top row) and *φ*_*eτ*_ (bottom row) of NSI parameters on the ability to distinguish between SI and NSI using LE + HE beam combination.

Finally, we also consider the case of CP violating NSI scenario. In Fig. 7, we go beyond the CP conserving NSI scenario considered so far and generalize Fig. 5 by considering non-zero NSI phases, *φ*_*eμ*_ and *φ*_*er*_. We show the ability of DUNE to discriminate between SI and CP violating NSI scenario by means of coloured oscillograms. The projection of the *χ*^2^ values at *φ*_*eμ*_ = 0 and *φ*_*eτ*_ = 0 (shown as dashed grey lines) in Fig. 7 correspond to the curves in Fig. 5 (red solid and dashed green curves in the bottom row, first column). The separation between scenarios in Fig. 7 is in accord with Fig. 5 which corresponds to the CP conserving NSI case as the separability is largest around *δ* ~ 0 and least around *δ* ~ ±*π*/2. For CP nonconserving NSI scenario (i.e. non-zero value of NSI phases *φ*_*eμ*_ or *φ*_*eτ*_), the general observation is that the best sensitivity for SI-NSI separation is no-longer near *δ* ~ 0 but rather shifts to other values of *δ* (see Fig. 7 and Table 4). This inturn implies that there is a strong correlation between the NSI phases (*φ*_*eμ*_ or *φ*_*eτ*_) and *δ*.

**Table 4:** The value of *δ* at which best possible sensitivity is attained for the CP nonconserving scenario as a function of *φ*_*eμ*_ and *φ*_*eτ*_ corresponding to the right column of Fig. 7 for the combination LE (1 + 1) + HE (4 + 4).

*ϕ*_*eμ*_/*π*	*δ*/*π* (max)	Δ*χ*^2^ at *δ*/*π* (max)
−1.0	0.04	7.34
−0.75	−0.17	6.12
−0.5	0.33	7.47
−0.25	0.13	8.90
0	−0.08	9.13
0.25	−0.25	8.38
0.5	−0.5	7.16
0.75	0.29	7.04
1.0	0.04	7.34
***ϕ***_***eτ***_ **/** ***π***	***δ*** **/** ***π*** **(max)**	**Δ** ***χ*** ^**2**^ **at** ***δ*** **/** ***π*** **(max)**
−1.0	−0.25	9.22
−0.75	−0.13	7.48
−0.5	−0.04	7.24
−0.25	−0.04	7.75
0	−0.08	9.13
0.25	−0.08	10.74
0.50	−0.17	11.52
0.75	−0.21	10.99
1.0	−0.25	9.22

## Summary and Outlook

Deep Underground Neutrino Experiment (DUNE)^3^ is a long baseline experiment that provides an excellent opportunity to answer the yet unanswered questions in neutrino oscillation physics such as deciphering whether CP is violated in the leptonic sector, what the neutrino mass hierarchy is and which octant *θ*_23_ resides in. It is pertinent to note that subdominant new physics effects can mask some of the undetermined parameters and one needs to think of new ways to eliminate any source of confusion. We utilize experimental handles to be able to extricate physics scenarios.

Our insight to use higher energy beams to isolate physics scenarios relies on the fact that the effect due to a given new physics scenario (here, NSI) tends to leave prominent signatures in CP asymmetries at the probability level for higher values of energy ($E\gtrsim 5\,{\rm{GeV}}$). We use higher energy beam tunes in conjunction with the existing LE beam in order to tap the information at higher energies. By exploiting the tunability of the beam, we offer a potentially viable strategy which could lead to better identification and discrimination of the new physics effects.

We have demonstrated an important usefulness of the wide band beam that is being considered for DUNE. We show that it is plausible to have better separation of SI from NSI if we consider different combinations of beam tunes and run times. For the CP conserving NSI scenario, the results are depicted in Figs 5 and 6 and summarized in Tables 2 and 3. Instead of running in LE only mode, a particular mix of LE and ME/HE is close to optimal with neutrino only mode (see Tables 2 and 3) for the purpose of separation of physics scenarios. The results are independent of the choice of hierarchy. The results also indicate that LE + HE beam combinations give slightly better results than LE + ME combinations. As can be seen from Fig. 7, for the CP nonconserving NSI scenario, the value of *δ* at which best possible sensitivity is attained is different (see Table 4) in comparison to the CP conserving scenario. The main conclusion is that we can distinguish different scenarios at 3*σ*(5*σ*) level for almost all (~50%) values of *δ*. Though the present study is in the context of NSI, the strategy pointed out is very general and can be applied to a variety of new physics scenarios. We are currently expanding the study to include more new physics scenarios.
